# Prevalence of tobacco use among women: a cross sectional survey from a squatter settlement of Karachi, Pakistan

**DOI:** 10.1186/s13104-015-1455-7

**Published:** 2015-09-24

**Authors:** Nousheen Iqbal, Muhammad Irfan, Nida Ashraf, Safia Awan, Javaid A. Khan

**Affiliations:** Department of Pulmonary Medicine, Aga Khan University Hospital, Stadium Road, Karachi, Pakistan; Department of Medicine, Aga Khan University, Stadium Road, Karachi, Pakistan; Aga Khan University Hospital, Stadium Road, Karachi, Pakistan; Department of Medicine, Aga Khan University Hospital, Stadium Road, Karachi, Pakistan

**Keywords:** Tobacco, Women, Prevalence, Squatter settlement

## Abstract

**Background:**

While the prevalence of tobacco use has been slowly declining in the developed countries, rates have been steadily rising in the developing countries. This has led to a rapid rise in tobacco related lung diseases among women.

**Objective:**

Determine the prevalence of tobacco use (both smoking and smokeless) among women in an urban squatter settlement (Orangi Town) in Karachi, Pakistan.

**Methods:**

A cross-sectional survey was conducted on 19,325 females aged between 15 and 80 years in Orangi Town, an urban squatter settlement in Karachi, Pakistan. Modified questionnaire, developed by World Health Organization WHO and Global Adult Tobacco survey (GATS), was used in Urdu. A total of 16,987 women agreed to participate.

**Results:**

The mean age was 37.3 ± 9.8 years amongst whom 15,255 (89.80 %) were married, 9143 (53.82 %) admitted that at least one person uses tobacco in some form in their homes. The prevalence of smokeless tobacco was 42.25 % (7178). The prevalence of smoking tobacco was low as compared to smokeless tobacco i.e. 18.0 % (3058). Among smokers, 85.47 % (1789) admitted that they had tried to quit smoking during last 12 months but failed.

**Conclusion:**

Tobacco use among women in an urban squatter settlement is very high and alarming. Preventive and control measures against tobacco use are required in these communities.

## Background

T Tobacco use is the leading cause of preventable mortality and morbidity worldwide [1, 2]. According to the WHO, tobacco use presently accounts for 6 million deaths each year [3]. Despite the increased awareness of hazardous effects of tobacco, it’s use has risen amongst women. This has led to a dramatic surge in tobacco related lung diseases like lung cancer and chronic obstructive airway disease COPD amongst women. Tobacco can be used in different fashions, ranging from cigarette, pipe smoking and cigar to smokeless tobacco products, available in various forms and mixtures like Pan/Betel quid, Naswar, Chalia/supari and Gutka.

According to Asian Agricultural Report 2014, Asia Pacific region is the fastest growing tobacco market in the world, with 4 out the 10 rapidly growing being: Malaysia, Indonesia, Pakistan & Vietnam. According to the report of World Health Survey (2002–2003) carried out in Pakistan, the prevalence of any form of smoking tobacco amongst adults was 19.9 % (33.5 % for males and 6.2 % for females) [4] and 1.8 and 4.6 % for smoking and smokeless tobacco respectively amongst women in a nationwide survey conducted in 2012 [5]. In 2007, a study showed prevalence of smoking amongst Pakistani young adolescent female of 16.3 % [6]. The mean age for trying to smoke cigarettes was 15 years.

There is a rapid increase in both use of smokeless tobacco as well as its inhaled forms [7]. Smokeless tobacco is the leading cause of cancers of the head and neck. In Pakistan and other Asian countries it’s used in much more culturally acceptable forms like Hookah, Naswar, Betel Quid and Gutka. This is the reason why around 58 % of these cancers worldwide occur in South and South East Asia alone [8, 9] and they constitute a major burden in Pakistan. This necessitates that tobacco control strategies in Pakistan should be focused not only on cigarette smoking, but also on smokeless tobacco consumption. Level of education and poverty remain an important predictor of tobacco consumption among men and women [10]. Tobacco consumption is seen largely in low income poverty areas. There is a serious dearth of information regarding current prevalence of tobacco use in women especially amongst squatter settlements in Pakistan, where the prevalence is relatively higher than other parts of city due to low socio-economic status, So we planned to investigate the prevalence of tobacco use both smoking as well as smokeless form among adult women in an urban squatter settlement (Orangi Town) in Karachi, Pakistan in collaboration with The All Pakistan Women’s Association (APWA) and the National Alliance for Tobacco Control (NATC).

## Methods

A cross sectional survey was conducted during the period July 01, 2012 to December 31, 2012 in Orangi Town, which is an urban squatter settlement in Karachi, Pakistan. The approximate population is two million. A cross-sectional survey was carried out on nearly 19,325 females between the ages of 15–80 years. There were total of 16,987 respondents. Face-to-face in-house interviews were conducted and a modified questionnaire developed by WHO and Global Adult Tobacco survey (GATS) [11], was used in Urdu. A brief training session was conducted and APWA female health workers were educated and fully trained to conduct this survey, a verbal consent was taken prior to the beginning of interview. All the surveyors gave standardized verbal explanations regarding this interview. Each interview took about 5 min. The questionnaire included demographic characteristics, current smoking status, use of smokeless tobacco, awareness and usefulness of the warning sign on cigarette packs.

### Sampling strategy

A cluster sampling technique was employed to draw the required sample. The Federal Bureau of Statistics (FBS) has divided the Orangi town into 13 union councils. The distance between the clusters ensured that the overall estimates are not influenced by factors peculiar to one geographic area. Trained community health workers explained the purpose and objectives of the study. Using software EPI Info and assuming 15 % prevalence of smoking in the study population with 95 % confidence level and a bound on error of ±1 % the estimated sample size was 4898. For cluster sampling we need to incorporate the design effect. We calculated a design effect of 2.9. The sample size required after multiplying with design effect is: *n* = 4898 × *D* = 944 × 2.9 = 14,204. However, this sample size is inflated by 15 %, to account for non-responders, making the final target sample size of 16,400 women.

The study was approved by Ethical review comity of NATC and ethical review board of APWA.

The analysis was carried out on SPSS v. 15. Frequencies were calculated for smokeless, smoked tobacco, nonsmokers, number of people using tobacco at home and passive smoking. Mean and standard deviation was calculated for age.

## Results

From July 01, 2012 to December 31, 2012, a total of 19,325 inhabitants were approached and 16,987 agreed to participate. The mean age was 37.3 ± 9.8 (range 15–80) and 15,255 (89.80 %) of the respondents were married (Table 1).

**Table 1:** Demographics of the study population

Variables	Total number (n) 16,987	Percentages
Age		
<30	4570	26.9
30–45	7304	43.0
>45	5113	30.1
Marital status		
Married	15,255	89.80
Single	1189	6.99
Divorced	510	3.01
Widowed	33	0.20
Profession		
Working women	5662	33.33
House wife	11,325	66.67

It was discovered that about 53.82 % (9143) of the households had at least one or more member in their family using tobacco in one form or another. Primarily, the most common form of tobacco use amongst women was smokeless 42.25 % (7178), 18 % (3079) were using smokeless tobacco while 5 % (849) of women were using both. Out of 18 % (3079) women who smoked tobacco, 67.95 % (2093) smoked cigarettes, 26.43 % (814) smoked Sheesha and 5.62 % (173) smoked Huqqa (Table 2).

**Table 2:** Prevalence of tobacco use

Variables	Total number (n) 16,987	Percentages
Smokeless	7178	42.25
Smoking	3079	18
Mixed	849	5
No. of family members using tobacco at home		
0	6732	39.63
1	9142	53.82
2	99	0.58
>3	1014	5.97
Passive smoking	2054	12.1
Form of smoking tobacco	n = 3079	
Cigarette	2093	67.95
Sheesha	814	26.43
Huqqa	173	5.62
Smoking status		
Daily	2052	98
Sometimes	41	1.95

The prevalence of passive smoking was 12.1 %. A total of 2093 people who smoked cigarette, were asked the question whether they had tried to quit smoking over the last 12 months, 85.47 % (1789) admitted that they had tried to quit smoking but failed while 79.21 % (1658) revealed that they had received medical advice to quit smoking. It was seen that majority of women 74.55 % (12,664) had not read any information in the newspapers regarding the harmful effects of smoking. However, there was a unanimous agreement that the picture on the cigarette pack showing hazards of tobacco use does deter them from smoking. At the end of this interview education on the hazardous effect of tobacco both smoking and smokeless was also provided by community health worker and 99.9 % promised to quit this habit.

## Discussion

Our study is the largest study that has been done amongst women in squatter settlement (Orangi town) Karachi, Pakistan to determine the prevalence of tobacco use both smoking and smokeless. Although surveys have been conducted in past to determine the prevalence of smoking tobacco, no such studies have yet been conducted in Pakistan for smokeless tobacco. The study has revealed that smokeless tobacco use is growing and its prevalence has increased to 42.25 %. Smoking tobacco use has mounted up to 18 % as compared to the national household survey done in Pakistan 2012 where tobacco use among female was negligible [5]. A cross-sectional survey conducted in Madaripur, Bangladesh showed the prevalence of smokeless tobacco amongst women to be 25 % [12]. The use of other forms of tobacco amongst females prevailed more than cigarettes in the present study. This may be because of greater cultural acceptability of these tobacco products over cigarettes among females in this region [9]. Betel Quid is served to guests in social gatherings and wedding ceremonies. Women are gifted Pandaan as a part of their trousseau/dowry. According to the 2011 WHO Report on the Global Tobacco Epidemic, Pakistan scores very poorly in terms of compliance with smoke-free legislation for public places. Considering the prevalence of culturally acceptable forms of tobacco among females, it is a cause for concern that there is no regular monitoring of smokeless tobacco in the country, nor are there any health warnings on smokeless tobacco products [13]. Women and youth in the developing world have been the target for most of these tobacco advertising tactics and there is an increase in smoking prevalence amongst women in these countries [14]. In our study the prevalence of smoking tobacco is 18.11 % and passive smoking incidence 12.1 % which is also high compare to last surveys done [4, 5]. It has been shown that smoking restrictions at home not only reduce the overall exposure of tobacco, but it can also reduce the smoking practice in youth [15]. A study done in Liaquat University Hospital, Hyderabad in pregnant women showed peer pressure and depression to be the two major reasons for commencement of tobacco, especially smoking use, among women [16]. These figures reflect the dire need to strengthen public health efforts that curtail all forms of tobacco advertisement, sponsorship and marketing strategies as well as to enforce strict laws to ban the use of not only cigarette but also smokeless tobacco.

Our analysis revealed that 79.21 % had been advised by their doctor to quit smoking and 85.47 % of the subjects had attempted to quit smoking during the past 12 months but failed. Quitting smoking is not easy and services for helping smokers to quit smoking are also limited in Pakistan [13]. Among current smokers in the United States, 68.8 % report that they want to stop smoking altogether and the number of former smokers has increased compared to the current smokers in the past 12 years [17]. This highlights the importance of these services in Pakistan to help those individuals who really wants to quit as majority are willing and failed due to ineffective cessation programs.

All respondents showed very limited knowledge about hazards of smoking 74.55 % had not read any information in the newspaper regarding the harmful effects of smoking however there was also a unanimous agreement amongst the smokers that the pictorial warning on the cigarette pack discourage them from smoking. This is consistent with International Tobacco Control surveys which also revealed the greater potential of pictorial warnings over text-only warnings [18]. Though cigarette packs in Pakistan contain pictorial health warnings, smokeless tobacco products do not. A study done in Canada revealed that pictorial health warnings on smokeless tobacco products were more effective in reducing its appeal [19]. These effects would also be more profound in countries like Pakistan. Currently Pakistan is facing double burden with communicable as well as non-communicable diseases (NCD) causing morbidity and mortality. According to estimation, nearly 54.9 % of the deaths in the country are caused by NCDs [20] and tobacco use is one of the biggest preventable risk factor.

### Limitations

Our study is largest study but there are certain limitations, first our survey mainly done at home during day time so majority were married, second we are failed to address the different form of smokeless tobacco as there were so many products available with different names, third ethnicity was not asked in this survey. As it was a questionnaire-based study, also introduces an element of recall bias. Finally, the data are based on self-reporting and their responses pertaining to smoking and smokeless tobacco were not biologically validated.

## Conclusion

So in conclusion tobacco use amongst women in an urban squatter settlement is alarmingly high. But good thing is majority of women are willing to quit this habits. Given the high morbidity and mortality associated with tobacco consumption, regulatory authorities need to enforce firm laws that ban the advertisement and promotion of all forms of tobacco. Current law in Pakistan only covers smoking tobacco, there is no law on smokeless tobacco and there is a need of law and legislation on smokeless tobacco too. They should also focus on having pictorial health warnings on smokeless tobacco products, the prevalence of which is higher amongst women with lower literacy rate. Meanwhile, there should also be a nationwide campaign in educating the masses against the dangers of not only smoking but also against smokeless tobacco, doctors and the media should play their roles in creating awareness and spreading information regarding its ill effects. Effective cessation programs are also necessary as majority is willing to quit. In last we recommend periodical survey and interventional studies in this high risk population to stop this tobacco epidemic.
